# AI is revolutionizing biomedical research, but are there any negatives?

**DOI:** 10.17179/excli2024-7761

**Published:** 2024-10-08

**Authors:** Bor Luen Tang

**Affiliations:** 1 Department of Biochemistry, Yong Loo Lin School of Medicine, National University Health System, National University of Singapore, 8 Medical Dr, Singapore 117596, Singapore

## ⁯

In his letter, Quintans-Júnior commented on the apparently unethical use of artificial intelligence (AI) in regional conflicts and warfare, and fear that unethical use of AI in medical robotics could bring harm to patients and users (Quintans-Júnior, 2024[8]). These worries are not unfounded. With further relevance to biomedicine, despite the exponential growth of AI use, the torrent of technical optimism and all the socioeconomic expectations of how AI could revolutionize the field, lingering concerns on potential risks remain. Here, I highlight two areas or aspects in biomedical *research* where limitations or issues associated with the extensive use of AI are beginning to emerge.

The first of which pertains to data generation and analysis. While the use of AI has greatly increased the generation of data, which in most reported cases have helped to enhance sensitivity and confidence in performing analytical diagnostics and in drawing of inferences, the question is whether we truly understand what this enormous amount of data actually means and what are its limits. Messeri and Crockett (2024[7]) have recently pointed out that AI's appeal comes from promises to improve productivity and objectivity, but at the same time AI also makes us vulnerable to illusions of understanding. Such illusions might “obscure the scientific community's ability to see the formation of scientific monocultures, in which some types of methods, questions and viewpoints come to dominate alternative approaches, making science less innovative and more vulnerable to errors”. As such the authors cautioned that “proliferation of AI tools in science risks introducing a phase of scientific enquiry in which we produce more but understand less” (Messeri and Crockett, 2024[7]). In other words, generating more of the same type of data so that these could be used for AI training could sideline new ways of think and finding innovative solutions to biomedical problems. In simply focusing on getting more refined image analysis or more omics datapoints from liquid biopsies that would feed machine learning pipelines in the hope of increasing the diagnostic precision or disease staging by a few percentage points, would we be ignoring novel, non-AI based approaches in tackling a devastating disease? 

An added perspective on data generation and analysis comes with the tremendous success in using powerful computational and machine leaning approaches to solve difficult problems, such as protein structure prediction (Jumper et al., 2021[4]). Commenting on the groundbreaking feat by AlphaFold2, Listgarten outlines the reasons why protein structure prediction was ultimately possible with supercomputing power and machine learning with the wealth of existing protein sequence and structural data (Listgarten, 2024[5]). However, “… the most interesting and impactful questions may not yet be formulated at all, let alone in a manner suitable for machine learning, or with existing suitable data, or even a way to readily generate suitable data for machine learning…”. Even limiting oneself to protein structure analysis, “… many important unsolved questions remain, primarily those of conformational dynamics and contextual effects, which will undoubtedly require yet more data to effectively tackle”. It is clear that feeding the same type of data into machine learning would only take us a certain distance. To go beyond would require innovation in acquiring novel and likely different types of data/pointers. 

We should therefore contemplate on the limits of what AI machine learning and recursive data feeding could achieve and spare some thoughts on how to step outside any potential limits of AI-based data analysis. In this regard, another note of caution was also made by a recent finding on AI-generated data. Generative AI could of course generate more data that could provide a larger analytic base. However, it was shown that for LLMs as well as variational autoencoders (VAEs) and Gaussian mixture models (GMMs), indiscriminate use of model-generated content in training causes irreversible defects that result in what is termed a “model collapse”, with a destructive erosion of output quality (Shumailov et al., 2024[9]). Although there will be ways around this, it would serve well as a reminder of the current limits of AI.

A second emerging issue pertains to the reporting, or publication, of biomedical research. Freely available generative AI or large language model (LLM)-based chatbots such as GPT3.5 and Gemini have made the writing of manuscripts from scratch so much easier and faster, and this has unsurprisingly led to their abuse. One estimation showed that in 2023 at least 60,000 papers (slightly over 1 % of all articles) were LLM-assisted (Gray, 2024[2]). Most journals and publishers do not forbid the use of AI in writing papers but have stated in their publishing guidelines or rules that such uses should be properly declared. There are two related issues associated with the use of AI in writing academic papers. The first concerns AI-based plagiarism, or AIgiarism, broadly define as a human author using AI-generated text or figures verbatim or with only cosmetic changes, without declaring the involvement of AI as such. I have argued elsewhere that AIgiarism is a form of bypass plagiarism, which facilitates the potential propagation of factual and interpretive errors (because LLMs tend to hallucinate) as well as biases (associated with the training datasets), which undermines knowledge acquisition and understanding (Tang, 2023[10]). It should be clear that the responsibility for errors incurred when human authors use AI-generated materials in papers lies squarely on the shoulders of the former, whose intention of taking shortcuts have inadvertently backfired.

The second related issue is more macroscopic in nature. Influenced by the publish or perish culture or under the delusion that more publications to one's name would effectively propel one's career forward, some have resorted to beefing up their publication list by writing papers even outside their actual areas of expertise with the help of LLMs. Before the advent of ChatGPT and other LLM-based chatbots, there are already a number of very productive scientists that publishes a paper every few days (Ioannidis et al., 2018[3]). However, there is now a surge in the number of such hyper prolific authors (Conroy, 2024[1]). While it is theoretically possible for a scientist endowed with large amounts of funding and a big team of coworkers and collaborators to be hyper-productive, hyper-productivity is unfortunately also associated with a more sinister trade, namely paper mills. With the LLM chatbots, papermills could now churn out fake research materials in the form of refined text and figures that would go quickly and readily into fabricated manuscripts for sale (Liverpool, 2023[6]). There is quite a large market for the latter in those that rely on a publication track record for a career step-up, as well as the large number of predatory journals that would publish these rather indiscriminately. Both AI-generated errors in papers and wholly fabricated papers produced using AI would damage science and academic publishing and should thus be checked and banished. 

The above limitations and issues on AI in biomedical research discussed are perhaps only the tip of the iceberg. This takes nothing away from the benefits of using AI and does not mean that we should be afraid of using AI in biomedical research. However, it does suggest that we need to exercise prudence with at least some aspects of AI-based work.

## Conflict of interest

The author declares no conflict of interest.
